# Transmission Risk from Imported *Plasmodium vivax* Malaria in the China–Myanmar Border Region

**DOI:** 10.3201/eid2110.150679

**Published:** 2015-10

**Authors:** Duoquan Wang, Shengguo Li, Zhibin Cheng, Ning Xiao, Chris Cotter, Jimee Hwang, Xishang Li, Shouqin Yin, Jiazhi Wang, Liang Bai, Zhi Zheng, Sibao Wang

**Affiliations:** Chinese Center for Disease Control and Prevention, Shanghai, China (D. Wang, N. Xiao);; World Health Organization Collaborating Centre for Tropical Diseases, Shanghai (D. Wang, N. Xiao);; National Center for International Research on Tropical Diseases, Shanghai (D. Wang, N. Xiao);; Tengchong County Center for Disease Control and Prevention, Tengchong, China (S. Li, X. Li, S. Yin, J. Wang);; Peking Union Medical College, Beijing, China (Z. Cheng, Z. Zheng);; University of California, San Francisco, California, USA (C. Cotter, J. Hwang);; Centers for Disease Control and Prevention, Atlanta, Georgia, USA (J. Hwang);; Chinese Academy of Sciences, Shanghai (L. Bai, S. Wang)

**Keywords:** malaria, vector-borne infections, parasites, imported malaria, *Plasmodium vivax*, vulnerability, receptivity, secondary infection, China–Myanmar border, *Anopheles sinensis*

## Abstract

Malaria importation and local vector susceptibility to imported *Plasmodium vivax* infection are a continuing risk along the China–Myanmar border. Malaria transmission has been prevented in 3 border villages in Tengchong County, Yunnan Province, China, by use of active fever surveillance, integrated vector control measures, and intensified surveillance and response.

A sharp increase in imported malaria cases has made preventing reintroduction of malaria in China a major challenge (*1*). High importation risk from Myanmar, where malaria is endemic, and wide distribution and abundance of malaria vectors in the China–Myanmar border region sustain risk for secondary infections among local populations. Tengchong County in Yunnan Province, bordering Myanmar, reported the highest number of imported malaria cases during 2010–2014 in China. A recent field survey indicated secondary transmission from imported *Plasmodium vivax* malaria in this region (*2*). To inform malaria elimination efforts in the region, we assessed local vectorial capacity and evaluated risk for secondary infections arising from malaria importation.

## The Study

Three villages (Manduo, Luoping, and Tuofeng; Figure) in Tengchong County, located in the westernmost part of Yunnan Province, were selected for study because of their 2011–2013 malaria incidence, ecologic features related to malaria transmission (i.e., altitude and proportion of land used for rice cultivation), and housing and economic status. The villages range in altitude from 1,276 to 1,893 meters and have 1,115 households and a population of 4,904 (detailed methods in Technical Appendix).

**Figure:**
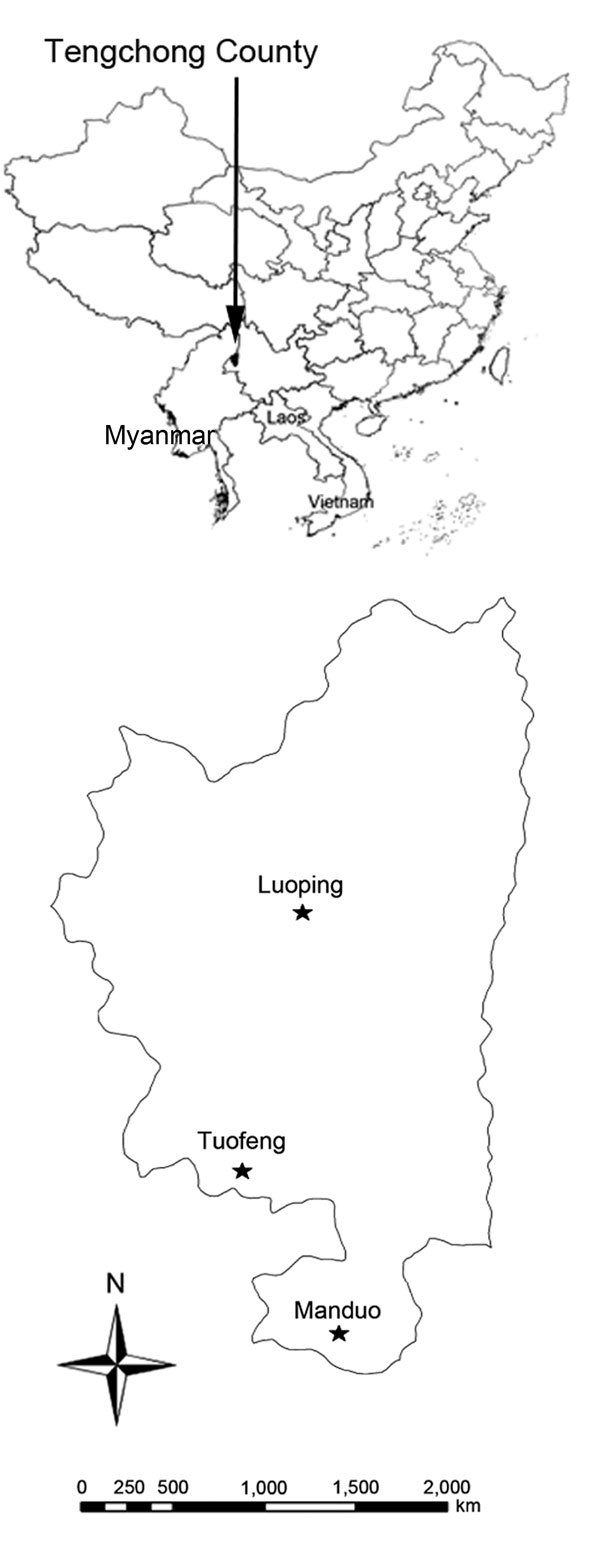
Location of 3 villages in Tengchong County, Yunnan Province, China, in which study of transmission risk from imported *Plasmodium vivax* malaria was conducted. Inset shows location of Tengchong County along the China–Myanmar border.

During 2011–2013, a total of 24 *P. vivax* malaria cases were reported from the study villages to the Chinese Information System for Disease Control and Prevention; all were classified as imported (Technical Appendix), as determined by patients’ travel history (*3*). All patients were adult men 18–49 years of age, and most worked in business or mining. Patients were radically cured with a regimen of chloroquine and primaquine, as recommended by national treatment guidelines. Malaria vulnerability or importation risk (i.e., incidence of imported malaria cases per 1,000 population per year) averaged 1.6 cases in the study area during 2011–2013 (Technical Appendix Table 1). The average interval between symptom development and diagnosis of malaria was 2.4 days; average interval between diagnosis and treatment was 1.5 days (Table 1). All cases were reported within 1 day and investigated within 3 days (Table 1).

**Table 1:** Malaria case management and response in 3 villages in the China–Myanmar border region, 2011–2013

Village	No. cases	Days from illness onset to diagnosis	Days from diagnosis to treatment	Reported within 1 d, %	Investigated within 3 d, %	Febrile, screened within 7 d, no.	Additional cases identified, no.
Manduo	8	3.2	1.6	100	100	124	0
Luoping	11	1.8	1.5	100	100	224	0
Tuofeng	5	2.2	1.5	100	100	99	0
Total	24	2.4	1.5	100	100	447	0

Health workers performing active fever surveillance during the 2013 transmission season (May–September) visited each house at 2-week intervals, conducted a total of 7,680 household interviews and 38,960 interviews with village residents, and collected 399 blood samples from persons with history of fever during the previous 2 weeks. A total of 268 (67.2%) samples were from local residents who reported no travel outside Tengchong County within the past 2 weeks; 131 were from mobile populations reporting travel. *P. vivax* isolates were detected by microscopy and PCR in 10 (7.6%) persons in the mobile population; no malaria infection was found in local persons (Technical Appendix Table 2).

To estimate human biting rates, mosquitoes were collected in each study village every 2 weeks by using volunteer outdoor human-landing catches during May–September 2013 (Technical Appendix). A total of 5,576 mosquitoes were caught; most (95%) were *Anopheles sinensis* mosquitoes. The average number of mosquitoes landing on a single person per night (human landing rate) was 2.7 (Table 2; Technical Appendix Table 3). The ratio of parous to nulliparous mosquitoes (multiparous ratio) of the *An. sinensis* mosquitoes tested for the villages of Manduo, Luoping, and Tuofeng was 0.52, 0.54, and 0.61, respectively (Technical Appendix Table 4). The average human blood index (i.e., proportion of tested mosquitoes with ingested human blood) was 3.8% in the surveillance areas (Table 2; Technical Appendix Table 5). Average vectorial capacity (i.e., expected number of new human infections from 1 infected person within 1 day, assuming all mosquitoes with sporozoites are potentially infective) of *An. sinensis* mosquitoes was 0.02 (Table 2), indicating that ≈50 cumulative days would be needed for transmission of malaria from an infected person to another person, assuming that all female mosquitoes biting malaria-infected persons become infected and transmit. The proportion of tested field-caught *An. sinensis* mosquitoes found to have ingested human blood (i.e., blood feeding rate) was 15.6% (31/199). The proportion of infected mosquitoes was 16.1% (5/31), the rate of susceptibility of local *An. sinensis* mosquitoes to imported *P. vivax* infection in this study.

**Table 2:** Vectorial capacity of *Anopheles sinensis* mosquitoes in 3 villages in the China–Myanmar border region, May–September 2013*

Village	Human landing rate†		Human blood index‡	Mosquito biting habits§	Daily survival rate¶	Days of sporogonic development#	p^n^**	Survival, d††	Receptivity‡‡
Manduo	1.2		0.04	0.02	0.8	12.4	0.1	5.4	0.01
Luoping	4.9		0.04	0.02	0.8	14.0	0.09	5.7	0.05
Tuofeng	2.0		0.03	0.01	0.9	23.3	0.04	7.2	0.01
Total	2.7		0.04	0.02	0.9	16.6	0.08	6.1	0.02

*Vectorial capacity, expected number of new human infections from 1 infected person within 1 day, assuming all mosquitoes with sporozoites are potentially infective. HBI, human blood index (proportion of tested mosquitoes having ingested human blood. †Average number of mosquitoes landing on a single person per night (*ma*). ‡Proportion of tested mosquitoes having ingested human blood. §Human blood index divided by days needed to complete gonotrophic cycle (cycle of taking a blood meal and laying eggs). ¶Probability (*p*) of a mosquito surviving 1 whole day. #Time (*n*) needed for parasites to complete development from ingested gametocytes during blood meal to sporozoites in salivary glands, when parasites are transmissible to humans. ** Fraction of infected mosquitoes after duration of sporogony. ††Duration of vector's life, in days, after surviving the extrinsic incubation period, calculated as the negative logarithmic reciprocal of the daily survival rate: 1/–ln(*p*).  ‡‡Expressed by the vectorial capacity index: *ma*^2^(*p^n^*/–ln(*p*)).

## Conclusions

*An. sinensis* mosquitoes were the main local vector for *P. vivax* and were widely distributed throughout this study area. The vector’s susceptibility to imported *P. vivax* infection indicates potential for *P. vivax* malaria to be sustained in this region. Although a recent field survey showed secondary transmission from patients with imported *P. vivax* malaria in other villages in Tengchong County (*2*), we identified no secondary infections in the study villages.

*An. sinensis* mosquitoes are the most widely distributed malaria vector in China but have relatively low susceptibility to parasites compared with other malaria vectors (*4*). We confirmed that *An. sinensis* mosquitoes were susceptible to imported *P. vivax* infection; however, vectorial capacity of *An. sinensis* mosquitoes was lower than it was during the 1990s (0.05) in the same region (*5*) and much lower than reported in central China (0.3) in the 1980s (*6*). The reduced vectorial capacity in the study region since the 1990s is likely attributable to the change in the dominant malaria vector species. Historically, *An. kunmingensis* mosquitoes were the main malaria vector in Tengchong County and accounted for 77% of total malaria vector density; its vectorial capacity (0.3) was ≈10 times higher than that of *An. sinensis* mosquitoes (0.03) in the 1980s (*7*). Few *An. kunmingensis* mosquitoes (<5% of total malaria vectors) were captured in our study, likely because of extensive residual insecticide use and improved housing, which reduce contact with this mosquito and vectorial capacity. High coverage (>90%) of long-lasting insecticidal nets and indoor residual spraying has been achieved in southern China along the Myanmar border since implementation of the National Malaria Elimination Program in July 2010.

In early 2012, a real-time surveillance system and response strategy (“1-3-7”) was rolled out nationally (*8*) and substantially improved timeliness of malaria surveillance and response activities. During 2011–2013, all malaria cases in the study area were reported within 1 day and investigated within 3 days, and screening of persons with fever was conducted within 7 days. Other countries reporting few days (range 3.0–8.2 days) before testing and treatment of imported malaria reported similar results and no subsequent secondary infections (*9*–*11*). In our study, additional testing by PCR for those reporting fever found no subpatent infections (i.e., slight infections with low parasitemia), a finding consistent with a recent review that showed no difference between PCR and microscopy for detecting parasites in symptomatic persons. However, a large proportion of *P. vivax* infections were subpatent in a cross-sectional survey of the general population in China (*12*).

Rigorous evaluation of malaria elimination programs is essential for continuously improving the programs, targeting limited resources, and maintaining financial and political support. We used robust entomologic and epidemiologic metrics to assess malaria elimination in a border region. Many national malaria control programs lack capacity to conduct entomologic surveillance to assess vectorial capacity. Although our study shows evidence of successful malaria elimination, additional validated metrics to ascertain success are needed. Recent efforts to compare the basic reproductive rate (total number of malaria cases derived from 1 infective case and distributed by mosquitoes in the absence of immunity) for imported versus local malaria cases provide a more nuanced and stable metric for measuring malaria elimination (*13*). Because this region has a unique ecology and distinct mosquito species composition, our findings need further validation to determine whether they can be extrapolated to other areas of China’s border region (*14*).

**Technical Appendix.** Detailed study methods and additional tables summarizing data on imported malaria cases, active fever surveillance, and entomologic investigations for study of transmission risk from imported *Plasmodium vivax* malaria along the China–Myanmar border.
