# Supplementary material for: Transmission Risk from Imported Plasmodium vivax Malaria in the China–Myanmar Border Region
Source: Emerg Infect Dis. 2015 Oct;21(10):1861–4. doi: 10.3201/eid2110.150679 (PMC4593446; doi:10.3201/eid2110.150679)
Supplement: Supplementary file 1 — Technical Appendix. Detailed study methods and additional tables summarizing data on imported malaria cases, active fever surveillance, and entomologic investigations for study of transmission risk from imported Plasmodium vivax malaria along the China–Myanmar border. [file 15-0679-Techapp-s1.pdf]

Article DOI: http://dx.doi.org/10.3201/eid2110.150679

Transmission Risk from Imported *Plasmodium vivax* Malaria in the China–Myanmar Border Region

Technical Appendix

Methods

Study Areas

The 3 villages (Manduo, Luoping, and Tuofeng) in our study have an altitude range of 1,276–1,893 meters and a total of 1,115 households with a population of 4,904. This area belongs to a tropical rainy climate and can be divided into a rainy season from July–September and a dry season from October–May. The proportion of the terraced rice fields in the study area was 20%–30% of total land in use. The villages have many different ethnic groups: Han, Dai, and Lisu (dominant groups) and Tibetans, Bai, Aini, and persons with Burmese ancestry. The villages are largely undeveloped areas in the border region, and many people frequently cross back and forth into Myanmar for employment in the logging, mining, and farming industries.

Ethical Approval

Approval for the study was obtained from the ethical committee of the National Institute of Parasitic Diseases, China Centers for Disease Control and Prevention (World Health Organization Collaborating Centre for Malaria, Schistosomiasis and Filariasis), Shanghai, China, and written informed consent was obtained from participants. No specific permissions were required, and field studies involved no endangered or protected species.

Malaria Surveillance Data

Routine malaria surveillance data during 2011–2013 from the study villages were obtained from the Chinese Information System for Disease Control and Prevention. All passively detected malaria cases were classified as local or imported on the basis of travel history. Imported malaria was defined by the following 3 criteria that must be simultaneously met: 1) diagnostically confirmed malaria; 2) travel history to malaria-endemic areas outside China during the malaria transmission season; and 3) onset of malaria <1 month after returning to China (*1*). Since the launch of the National Malaria Elimination Program in July 2010, each case of malaria is required to be classified as local or imported, and the source of infection for each case should be investigated within 3 days according to the above criteria.

Study Participants

To monitor for malaria infection among mobile and local populations in the study areas, active case detection was implemented and targeted all populations through household visits every 2 weeks during the 2013 transmission season (May–September). Any persons, local or mobile, with history of fever in the past 2 weeks were tested for malaria. Local populations were defined as those residents with no travel history outside Tengchong County within the past 2 weeks; mobile populations reported travel.

Blood Sample and Detection

Thick and thin blood films for parasitologic diagnosis were taken from all persons who reported a history of fever in the past 2 weeks in the study areas. Blood films were stained by the standard Giemsa method, usually within 48 hours, but always within 96 hours. Examinations were performed by a senior technician from the local Provincial Center for Disease Control and Prevention and then checked by national experts for quality assurance of findings. All discordant results were reviewed together and then confirmed by PCR. Examination of >200 high-power oil immersion fields was required to verify a negative blood film. Dried blood spot samples obtained by filling 2 delineated circles (≈0.5 inch in radius) on a filter paper were thoroughly dried and kept refrigerated at 4°C before their delivery to the national laboratory for PCR processing. In this study, we applied a novel high-throughput PCR, Capture and Ligation Amplification PCR method (CLIP-PCR) (*2*), to detect the 18S rRNA of *Plasmodium* spp. in all dried blood spots. To accelerate the diagnostic efficiency and reduce costs, samples were tested with a matrix pooling strategy (*3*). At least 1 positive control and 1 negative control were included in each assay, and each sample was tested in duplicate.

Entomological Surveillance

To estimate human biting rates, mosquitoes were collected in each study village every 2 weeks by using volunteer outdoor human-landing catches during May–September 2013, according to methods recommended by the World Health Organization (*4*). All mosquitoes that landed on a volunteer were assumed to have bitten and were collected and identified by their morphologic features (*5*). Three villages were sampled simultaneously, and mosquito collectors were rotated among the 3 study sites to eliminate bias. The mosquito-biting rate was estimated by the number of certain species of mosquitoes caught per person per night in each village. To estimate a human blood index, blood-engorged *Anopheles* spp. were collected from different types of mosquito resting places (i.e., the bush, empty cow-sheds, small warehouses, and concrete bunkers) by using a US Centers for Disease Control and Prevention backpack aspirator (John W. Hock Co.,

Gainesville, Florida, USA); blood meals were identified by PCR (*6*). The human blood index was estimated by applying the unweighted mean of a selected portion of all collected samples collected from different types of mosquito resting places (*7*). A survey to determine multiparous mosquito ratios was conducted in the villages, where mosquitoes were dissected daily to determine their parity, following methods reported by Dong et al. (*8*). Half of the female mosquitoes collected were randomly selected and dissected after each collection. To estimate vector competence, female anopheline mosquitoes collected from the study areas were reared at 27°C (± 1°C) and 70%–80% relative humidity and were provided with a 10% (weight/volume) sucrose solution. Adult female mosquitoes were starved for 6 hours before infection by membrane feeding assay, as previously described (*2*). The mosquitoes were allowed to feed for 45 minutes on *P. vivax*–infected whole blood meals. Mosquitoes that had not fed on blood were removed within 24 hours. Mosquitoes were then maintained at 26°C and 70% (± 5%) relative humidity and were provided with a 10% sucrose solution. To estimate infectivity, the midguts of the vectors were dissected on day 7 after the blood feeding and stained with 0.1% (weight/volume) mercurochrome in phosphate-buffered saline; oocyst numbers per midgut were examined by microscopy.

Estimation of receptivity

The receptivity index or vectorial capacity (VC = *ma*2[*Pn*/-ln*P*]) is defined as the expected number of new infections per infective case per day (provided all mosquitoes with sporozoites are potentially infective); vectorial capacity was interpreted by Garret-Jones as the product of the man-biting rate (ma), biting habits (a), and longevity factor (P^n/-ln*P*) (*7*). Mosquito biting habits (a) are defined as the human blood index divided by the length of the gonotrophic cycle, on the basis of previous research from China. The daily survival rate (P) was calculated according to Qian et al (*8*). We calculated life expectancy values for *P. vivax*–infected *Anopheles* spp. by using methods outlined in a similar study (n = 105/[T-14.5]). In the equation, 105 is the number of days needed for the *P. vivax* sporozoite to mature in the *Anopheles* mosquito at the threshold

temperature of 14.5°C, and T is the local average atmosphere temperature during May–September 2013 (*5*).

References

1. Chinese Centers for Disease Control and Prevention. Technical scheme of malaria elimination [in Chinese]. 2011 [cited 2011 Sep 2]. http://www.chinacdc.cn/jkzt/crb/nj/jszl_2223/201109/U020110906536170416565.pdf

2. Cheng Z, Wang D, Tian X, Sun Y, Sun X, Xiao N, et al. Capture and Ligation Probe-PCR (CLIP-PCR) for Molecular Screening, with application to active malaria surveillance for elimination. Clin Chem. 2015;61:821–8. PubMed http://dx.doi.org/10.1373/clinchem.2014.237115

3. Smith DM, May SJ, Perez-Santiago J, Strain MC, Ignacio CC, Haubrich RH, et al. The use of pooled viral load testing to identify antiretroviral treatment failure. AIDS. 2009;23:2151–8. PubMed http://dx.doi.org/10.1097/QAD.0b013e3283313ca9

4. World Health Organization. Manual on practical entomology in malaria. part II: methods and techniques. Geneva: The Organization; 1975.

5. Ministry of Health Disease Prevention and Control Bureau. Handbook for malaria control and prevention. Beijing: People's Hygiene Publishing House Press; 2007.

6. Cheng Q, Cunningham J, Gatton ML. Systematic review of sub-microscopic P. vivax infections: prevalence and determining factors. PLoS Negl Trop Dis. 2015;9:e3413. PubMed http://dx.doi.org/10.1371/journal.pntd.0003413

7. Garrett-Jones C. Prognosis for interruption of malaria transmission through assessment of the mosquito's vectorial capacity. Nature. 1964;204:1173–5. PubMed http://dx.doi.org/10.1038/2041173a0

8. Qian H, Tang L, Cheng Y, Yang B. Preliminary estimation of malaria transmission potential in areas where *Anopheles sinensis* is the only vector [in Chinese]. Zhongguo Ji Sheng Chong Xue Yu Ji

Sheng Chong Bing Za Zhi [Chinese Journal of Parasitology and Parasitic Diseases]. 1994;12:265–7. PubMed

Technical Appendix Table 1. Number of malaria cases reported in 3 villages and annual parasite incidence rates, classified as imported or local cases, China–Myanmar border region, 2011–2013*

			Malaria cases by year, no. (API)							
			2011		2012		2013		2011–2013	
Village	Altitude, m	Population	Local	Imported	Local	Imported	Local	Imported	Local	Imported
Manduo	1,276	1,079	0	2 (1.9)	0	3 (2.8)	0	3 (2.8)	0	8 (2.5)
Luoping	1,752	2,249	0	6 (2.7)	0	2 (0.9)	0	3 (1.3)	0	11 (1.6)
Tuofeng	1,893	1,576	0	2 (1.3)	0	1 (0.6)	0	2 (1.3)	0	5 (1.1)
Total		4,904	0	10 (2.0)	0	6 (1.2)	0	8 (1.6)	0	24 (1.6)

*API, Annual parasite incidence (cases/1,000 persons/year). Source: Chinese Information System for Disease Control and Prevention.

Technical Appendix Table 2. Number of persons tested for malaria and those testing positive in 3 villages during the malaria transmission season, by local and mobile populations and testing method, China–Myanmar border region, 2013*

	Mobile Population			Local Population		
		Method of Testing			Method of Testing	
Village	No. tested	Microscopy, no. + (%)	PCR, no. + (%)	No. tested	Microscopy, no. + (%)	PCR, no. + (%)
Manduo	28	3 (10.7)	3 (10.7)	70	0 (0.0)	0 (0.0)
Luoping	69	5 (7.3)	5 (7.3)	152	0 (0.0)	0 (0.0)
Tuofeng	34	2 (5.9)	2 (5.9)	46	0 (0.0)	0 (0.0)
Total	131	10 (7.6)	10 (7.6)	268	0 (0.0)	0 (0.0)

*Testing resulted from active fever surveillance. Malaria transmission season in study area is May–September; no. +, number testing positive.

Technical Appendix Table 3. Average monthly human-landing rate *of Anopheles sinensis* mosquitoes in 3 villages during the malaria transmission season, China–Myanmar border region, 2013*

Village	May	June	July	August	September	Village Average
Manduo	0.7	1.2	3.5	0.7	0.0	1.2
Luoping	2.0	5.3	8.0	5.0	4.3	4.9
Tuofeng	1.7	4.3	2.6	1.6	0.0	2.0
Monthly Average	1.5	3.6	4.7	2.4	1.4	2.7

*Human landing rate = number of mosquitoes landing on a single person per night. Malaria transmission season in the study area is May–September.

Technical Appendix Table 4. Parity of *Anopheles sinensis* mosquitoes collected in 3 villages during the malaria transmission season, China–Myanmar border region, 2013*

Village	Test mosquitoes	Parous	Nulliparous	Parity Ratio	X^2 test among groups
Manduo	128	66	62	0.5	$X^2 = 6.23$ p-value = 0.10
Luoping	136	74	62	0.5	
Tuofeng	140	92	48	0.7	
Total average	135	77	57	0.6	

*Malaria transmission season in the study area is May–September.

Technical Appendix Table 5. Human blood–meal Identification of *Anopheles sinensis* mosquitoes in 3 villages during the malaria transmission season, China–Myanmar border region, 2013*

Village	Engorged mosquitoes tested, no.	Mosquitoes containing human blood, no. (%)	X^2 test among groups
Manduo	74	3 (4.1)	$X^2 = 0.14$ p-value = 0.9
Luoping	71	3 (4.2)	
Tuofeng	65	2 (3.1)	
Total	210	8 (3.8)	

*Malaria transmission season in the study area is May–September.
